# Integrative analysis of gene expression profiles reveals specific signaling pathways associated with pancreatic duct adenocarcinoma

**DOI:** 10.1186/s40880-018-0289-9

**Published:** 2018-04-27

**Authors:** Jun Li, Wenle Tan, Linna Peng, Jialiang Zhang, Xudong Huang, Qionghua Cui, Jian Zheng, Wen Tan, Chen Wu, Dongxin Lin

**Affiliations:** 1 Department of Etiology and Carcinogenesis, National Cancer Center/Cancer Hospital, Chinese Academy of Medical Sciences and Peking Union Medical College, Beijing, 100021 P.R. China; 2 Sun Yat-sen University Cancer Center, State Key Laboratory of Oncology in South China, Collaborative Innovation Center for Cancer Medicine, Guangzhou, Guangdong 510060 P.R. China; 3 Collaborative Innovation Center for Cancer Personalized Medicine, Nanjing Medical University, Nanjing, Jiangsu 210009 P.R. China

**Keywords:** Pancreatic cancer, GEO, Pathway network, *CKS2*

## Abstract

**Background:**

Pancreatic duct adenocarcinoma (PDAC) remains a major health problem because conventional cancer treatments are relatively ineffective against it. Microarray studies have linked many genes to pancreatic cancer, but the available data have not been extensively mined for potential insights into PDAC. This study attempted to identify PDAC-associated genes and signaling pathways based on six microarray-based profiles of gene expression in pancreatic cancer deposited in the gene expression omnibus database.

**Methods:**

Pathway network methods were used to analyze core pathways in six publicly available pancreatic cancer gene (GSE71989, GSE15471, GSE16515, GSE32676, GSE41368 and GSE28735) expression profiles. Genes potentially linked to PDAC were assessed for potential impact on survival time based on data in The Cancer Genome Atlas and International Cancer Genome Consortium databases, and the expression of one candidate gene (*CKS2*) and its association with survival was examined in 102 patients with PDAC from our hospital. Effects of *CKS2* knockdown were explored in the PDAC cell lines BxPC-3 and CFPAC-1.

**Results:**

The KEGG signaling pathway called “pathway in cancer” may play an important role in pancreatic cancer development and progression. Five genes (*BIRC5*, *CKS2*, *ITGA3*, *ITGA6* and *RALA*) in this pathway were significantly associated with survival time in patients with PDAC. *CKS2* was overexpressed in PDAC samples from our hospital, and higher *CKS2* expression in these patients was associated with shorter survival time. *CKS2* knockdown substantially inhibited PDAC cell proliferation in vitro.

**Conclusions:**

Analysis integrating existing microarray datasets allowed identification of the “pathway in cancer” as an important signaling pathway in PDAC. This integrative approach may be powerful for identifying genes and pathways involved in cancer.

**Electronic supplementary material:**

The online version of this article (10.1186/s40880-018-0289-9) contains supplementary material, which is available to authorized users.

## Background

Pancreatic ductal adenocarcinoma (PDAC), which accounts for approximately 90% of all pancreatic tumors [1], is the ninth leading cause of cancer-related deaths in China [2]. Due to the lack of effective methods for early diagnosis and therapy, pancreatic cancer has the second highest ratio of mortality to incidence (approximately 88%) among all types of cancer [3]. Microarray platforms have produced numerous datasets of gene expression profiles in pancreatic cancer, which are publicly available through the gene expression omnibus (GEO) of the National Center for Biotechnology Information [4]. Studies of such data have identified several genes associated with pancreatic cancer, but these datasets have yet to be mined extensively to identify genes and pathways linked to PDAC.

To identify additional genes that may be associated with PDAC, we mined six frequently cited profiles of genes expressed in pancreatic cancer that have been deposited in the GEO database. We used pathway network analysis to identify core pathways that may affect pancreatic cancer development and progression. Our analysis of genes differentially expressed in pancreatic cancer took into account expression quantitative trait loci [5], copy number variation [6, 7], and DNA methylation [8], all of which can affect gene expression levels.

## Methods

### Affymetrix microarray data

The following pancreatic cancer gene expression profiles were downloaded from the GEO database (http://www.ncbi.nlm.nih.gov/geo/): GSE71989, GSE15471, GSE16515, and GSE32676, which were obtained using an Affymetrix GeneChip^®^ Human Genome U133 Plus 2.0 Array; as well as GSE41368 and GSE28735, which were obtained using an Affymetrix Human Gene 1.0 ST Array.

### Data processing

We downloaded the probe-level data from the GEO database as CEL files, then we used the GCBI on-line analysis tool (http://www.gcbi.com.cn) to normalize and log_2_-transform the data. Genes differentially expressed between tumor samples and non-tumor samples were filtered by requiring a fold difference of at least two and a maximum false discovery rate of 0.05. Pathway analysis was performed using DAVID software (https://david.ncifcrf.gov) as described [9].

### Core pathway analysis

Pathways enriched for genes differentially expressed between tumor and non-tumor tissues were identified and connected in a pathway network to reveal relationships among the pathways. A pathway network was constructed using the GCBI on-line analysis tool, and for each pathway in the network, the number of upstream pathways (in-degree) and the number of downstream pathways (out-degree) were determined in order to assess the degree centrality of that pathway in the network. Greater degree centrality means that a pathway regulates or is regulated by more pathways, implying a more important role in the network.

### CKS2 analysis based on database mining

Copy number variation of *CKS2* in various types of cancer was analyzed using the cBioPortal database (http://www.cbioportal.org), and *CKS2* overexpression in cancer was analyzed using the Oncomine database (http://www.omcomine.org). *CKS2* expression levels in various cancer cell lines were analyzed using the CCLE database (https://portals.broadinstitute.org/ccle/home).

### PDAC cell culture

Human PDAC cell lines BxPC-3 and CFPAC-1 were purchased from the Cell Bank of Type Culture Collection (Chinese Academy of Sciences, Shanghai Institute of Biochemistry and Cell Biology). The human immortalized pancreatic duct epithelial cell line HPDE6-C7 was purchased from Biotechnology Company. All cell lines were characterized by DNA fingerprinting and tested for mycoplasma and found to be free of infection.

BxPC-3 and HPDE6-C7 cells were cultured in RPMI-1640 medium (Gibco, Thermo Fisher Scientific, USA) supplemented with 10% fetal bovine serum (FBS; Hyclone, USA), while CFPAC-1 cells were cultured in IMDM (Gibco, Thermo Fisher Scientific) supplemented with 10% FBS. All cell lines were grown without antibiotics in an atmosphere of 5% CO_2_ and 99% relative humidity at 37 °C.

### Survival analysis of key genes

SurvExpress [10] was used to analyze survival associated with key genes found in this study.

### Patient samples

Surgical biopsies of tumor tissues and paired non-tumor tissues from patients definitively diagnosed with PDAC based on histology were obtained from the Department of Hepatobiliary Surgery at Sun Yat-sen Memorial Hospital (Guangzhou, China). Tissues were preserved in liquid nitrogen immediately following surgical removal, paraffin-embedded and stored properly before use in the present study. None of the patients had received anticancer treatment before biopsy collection. Clinicopathology and follow-up data for patients are shown in Additional file 1: Table S1.

### Western blotting for CKS2

Total protein (20 μg) was extracted from cells or patient tissue samples, resolved by SDS-PAGE and transferred to PVDF membranes (Millipore, Germany). Membranes were incubated with antibodies against CKS2 (ab155078; Abcam, Cambridge, UK) or β-actin (ab8227; Abcam), then with horseradish peroxidase-conjugated secondary antibody from the Phototope-HRP Western blot detection kit (Cell Signaling Technology, USA). Protein bands were visualized using the components in this kit.

### CKS2 knockdown in PDAC cells

Short interfering RNA (siRNA) targeting *CKS2* and non-targeting control siRNA (Additional file 2: Table S2) were purchased from GenePharma (Suzhou, China). PDAC cells were transfected with siRNA using Lipofectamine 2000 (Life Technologies, USA) according to the manufacturer’s instructions.

### Proliferation of PDAC cell lines

Cells were seeded into 96-well plates (2 × 10^3^ cells in 100 µL per well) and cultured for certain periods. Then cell viability was measured using a CCK-8 assay (Dojindo, Japan). Samples were analyzed as six replicates per experiment, and three independent experiments were performed.

### RNA extraction and qRT-PCR analysis

Total RNA from pancreatic cancer cell lines was extracted using TRIzol regent (Invitrogen, USA). First-strand cDNA was synthesized using the PrimeScript™ RT reagent Kit (TaKaRa, Japan) and amplified by qRT-PCR on an ABI Prism 7900 sequence detection system (Applied Biosystems) using SYBR Green. Sequences of gene-specific primers are listed in Additional file 3: Table S3.

### Statistical analyses

All statistical analyses were performed using Graphpad Prism 5.0 and *P *< 0.05 was considered significant. Differences between two groups were assessed for significance using Student’s *t* test. Kaplan–Meier survival estimates were plotted, and inter-group differences were assessed using the log-rank test.

## Results

### Genes differentially expressed in pancreatic cancer based on integrative analysis of GEO datasets

**Fig. 1:**
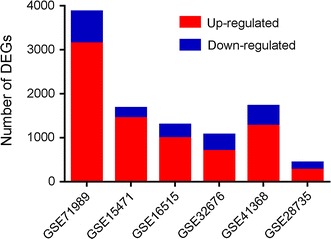
Genes differentially expressed between pancreatic cancer tissues and normal tissues. We analyzed differentially expressed genes in six GEO microarray datasets. Up-regulated: the number of genes whose expression levels were higher in pancreatic cancer tissues than in normal tissues. Down-regulated: the number of genes whose expression levels were lower in pancreatic cancer tissues than in normal tissues. *GEO* Gene Expression Omnibus, *DEG* differentially expressed gene

We examined six gene expression profiles from the GEO database (Additional file 4: Table S4) in order to identify genes differentially expressed between PDAC and normal tissues. One sample each from GSE15471 to GSE28735 were excluded from further analysis because they did not meet quality controls. Analysis of the remaining samples indicated a larger number of genes up-regulated in PDAC than down-regulated (Fig. 1).

### The “pathway in cancer” is the top core pathway in pancreatic cancer

**Fig. 2:**
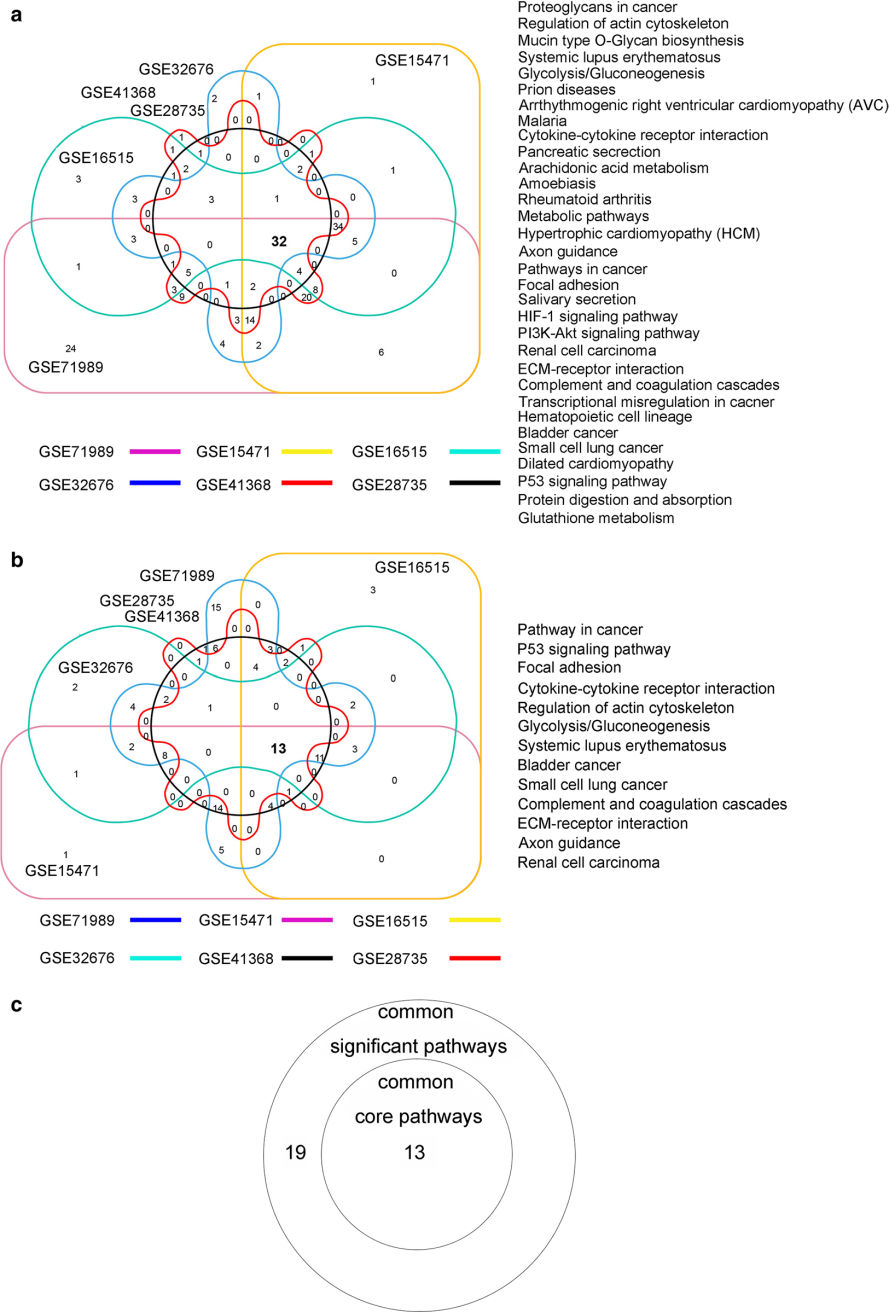
Core pathway analysis of six GEO datasets. **a** Overlap of significant pathways in each GEO dataset used in this study revealed 32 common significant pathways. *Left*, Venn diagram of the result. *Right*, names of common significant pathways. **b** Overlap of the core pathways of each GEO dataset used in this study revealed 13 common core pathways. *Left*, Venn diagram of the result. *Right*, names of common core pathways. **c** Relationship between the common significant pathways and common core pathways

Genes differentially expressed between PDAC and non-tumor tissues in each of the six profiles were assembled into functional pathways. As expected, the pathways that were enriched in differentially expressed genes varied among the six profiles (data not shown). We identified 32 significant pathways common to the six profiles (Fig. 2a). We also used network analysis to identify pathways common to the six profiles that were likely to be most important in PDAC, which led to 13 common core pathways (Fig. 2b): pathway in cancer, p53 signaling pathway, focal adhesion, cytokine–cytokine receptor interaction, regulation of actin cytoskeleton, glycolysis/gluconeogenesis, systemic lupus erythematosus, bladder cancer, small cell lung cancer, complement and coagulation cascades, ECM-receptor interaction, axon guidance, and renal cell carcinoma. Interestingly, all 13 common core pathways were among the 32 common significant pathways (Fig. 2c). Details of the 13 common core pathways in each profile are shown in Table 1. The pathway in cancer (KEGG ID: 05200) was the top significant pathway, with a total degree centrality of 116. This suggests that the pathway in cancer may play an important role in PDAC development and progression.

**Table 1:** Summary of 13 common core pathways associated with pancreatic cancer in six GEO microarray datasets

Pathway ID	Pathway name	GSE71989		GSE15471		GSE16515		GSE32676		GSE41368		GSE28735		Total degree
		Outd	Ind	Outd	Ind	Outd	Ind	Outd	Ind	Outd	Ind	Outd	Ind	
05200	Pathway in cancer	28	0	21	0	15	0	23	0	21	0	8	0	116
04115	P53 signaling pathway	2	16	2	12	2	6	1	13	1	11	0	3	69
04510	Focal adhesion	8	7	7	5	6	4	6	5	7	6	3	4	68
04060	Cytokine-cytokine receptor interaction	0	16	0	14	0	8	0	9	0	13	0	4	64
04810	Regulation of actin cytoskeleton	3	9	3	8	3	6	3	7	3	9	1	2	57
00010	Glycolysis/Gluconeogenesis	2	14	0	6	3	11	0	4	2	10	1	3	56
05322	Systemic lupus erythematosus	8	0	7	0	6	0	6	0	8	0	2	0	37
05219	Bladder cancer	6	1	5	1	5	1	5	1	4	1	1	1	32
05222	Small cell lung cancer	5	1	5	1	5	1	4	1	4	1	3	1	32
04610	Complement and coagulation cascades	1	7	0	6	0	2	0	2	1	6	0	1	26
04512	ECM-receptor interaction	1	3	1	3	1	3	1	3	1	3	1	3	24
04360	Axon guidance	3	0	3	0	3	0	3	0	3	0	2	0	17
05211	Renal cell carcinoma	4	1	2	1	1	1	2	1	2	1	0	1	17

*Outd* outdegree, the number of downstream signaling pathways connected to the node signaling pathway;* Ind* indegree, the number of upstream signaling pathways connected to node signaling pathway; Total degree: outdegree + indegree

### Association of BIRC5, CKS2, ITGA3, ITGA6, and RALA with survival time in PDAC

**Fig. 3:**
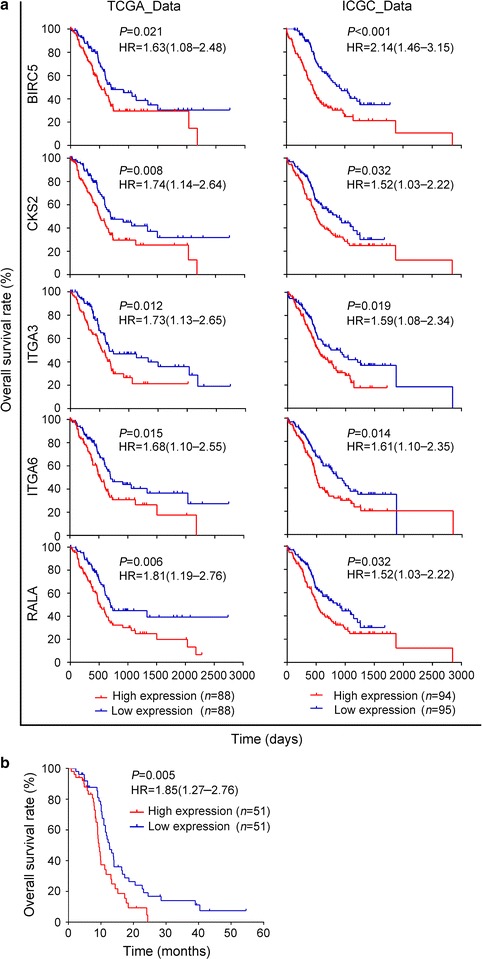
Identification of an association of *BIRC5*, *CKS2*, *ITGA3*, *ITGA6* and *RALA* with survival time of patients with PDAC. **a** Kaplan–Meier survival curves of *BIRC5*, *CKS2*, *ITGA3*, *ITGA6* and *RALA* in The Cancer Genome Atlas (Internal ID: 811) or International Cancer Genome Consortium database (Internal ID: 812). Survival data were obtained from the SurvExpress database. **b** Association of *CKS2* expression with survival time in a cohort of 102 patients with PDAC at our hospital

To characterize the function of the pathway in cancer in PDAC, we analyzed the differentially expressed genes of this pathway in each of the six expression profiles. A total of 137 genes in this pathway were found to be differentially expressed in pancreatic cancer across all six datasets. Mining of The Cancer Genome Atlas (Internal ID: 811) and the International Cancer Genome Consortium database (Internal ID: 812) provided survival data connected with these 137 genes (Additional file 5: Table S5). We identified five genes that have been associated with patient survival time: baculoviral IAP repeat containing 5 (*BIRC5*), CDC28 protein kinase regulatory subunit 2 (*CKS2*), integrin subunit alpha 3 (*ITGA3*), integrin subunit alpha 6 (*ITGA6*), and RAS-like proto-oncogene A (*RALA*) (Fig. 3a). Patients with high expression of these five genes showed shorter survival. Consistent with these database results, we examined *CKS2* expression in 102 patients with PDAC at our hospital and found that higher expression was associated with shorter survival (Fig. 3b).

### Overexpression of CKS2 in PDAC

**Fig. 4:**
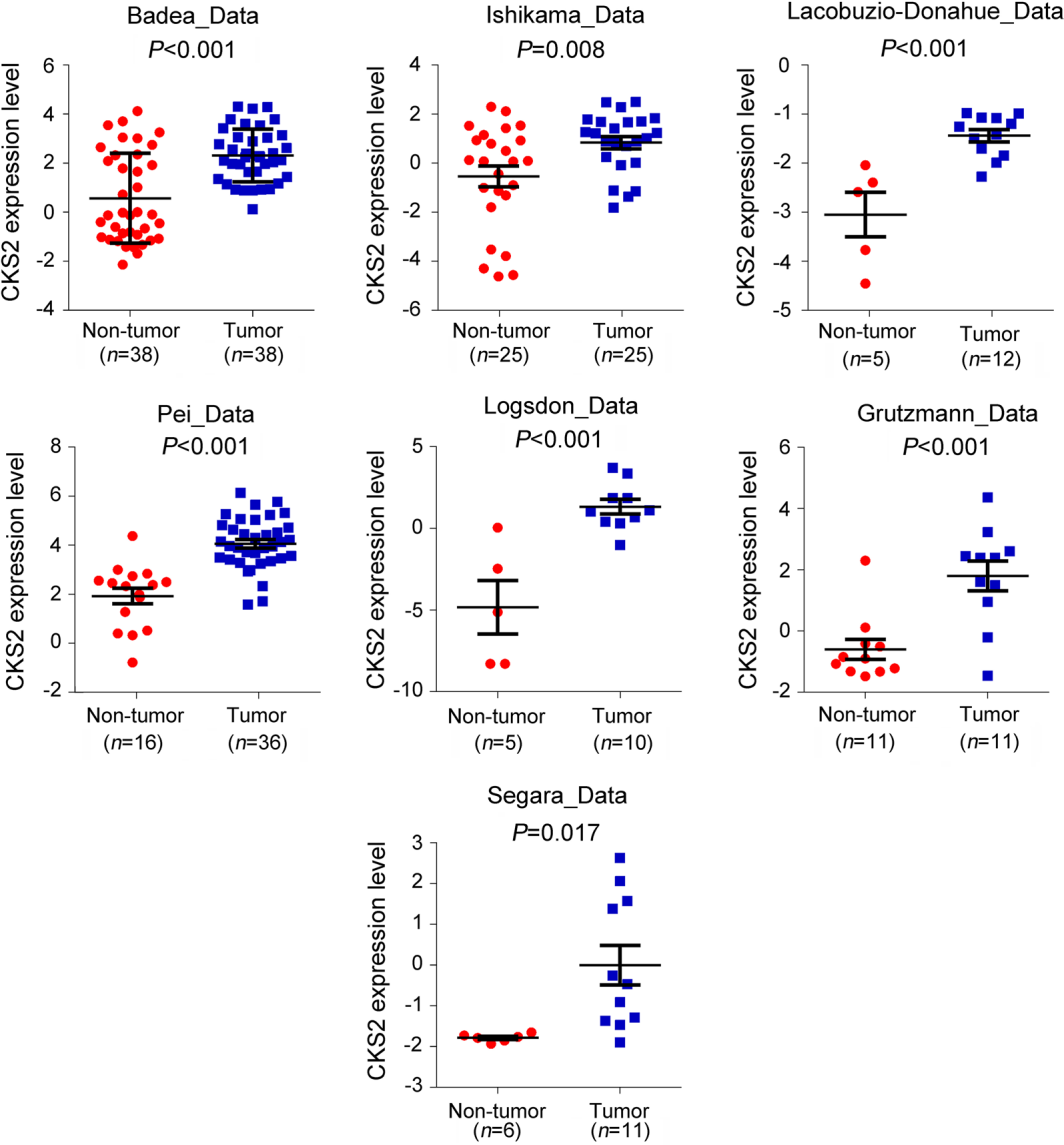
Overexpression of *CKS2* in PDAC. *CKS2* mRNA levels were significantly higher in PDAC tissues than in normal tissues according to the Oncomine database. Red color indicates *CKS2* expression levels in normal pancreatic tissues, while blue color indicates *CKS2* expression levels in pancreatic cancer

**Fig. 5:**
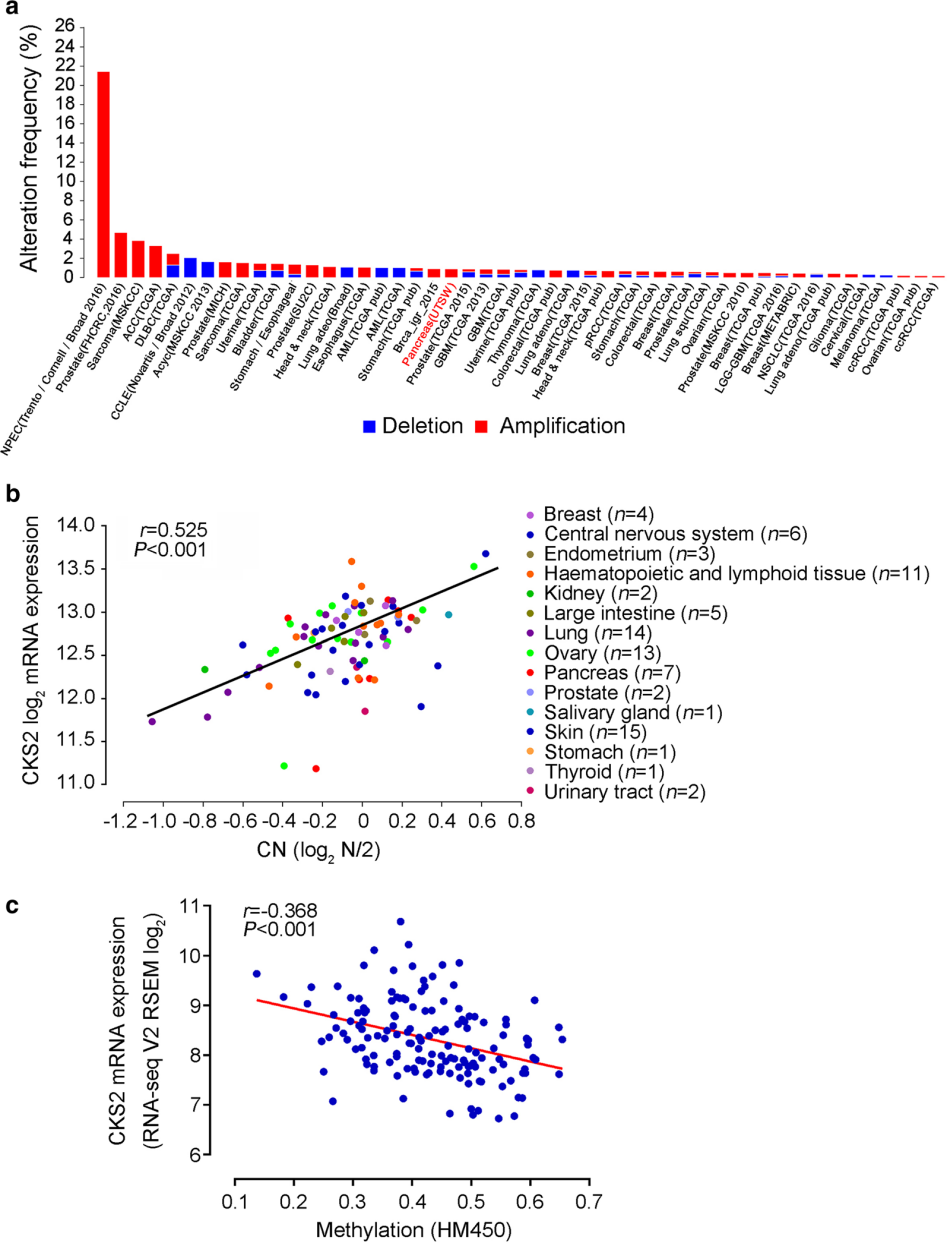
Genomic alterations of *CKS2* in human cancers. **a** Summary of copy number variations of *CKS2* across 49 cancer genomic studies (http://www.cbioportal.org). The results are displayed as a histogram of the frequencies of *CKS2* copy numbers across cancer studies. **b** Correlation between *CKS2* copy number and *CKS2* expression level in 87 cancer cell lines derived from 15 types of human cancer (http://portals.broadinstitute.org/ccle/home). Pearson’s correlation coefficient (*r*) is shown. **c** Correlation of *CKS2* expression and DNA methylation in 149 pancreatic cancer tissues in The Cancer Genome Atlas database. Pearson’s correlation coefficient (*r*) is shown

To determine which of these survival-related genes may be most important, we analyzed the fold-difference in the expression of each gene between PDAC and non-tumor tissues in the six expression profiles (Table 2). *CKS2* showed the largest difference in expression, being up-regulated 4.82-fold in PDAC. Overexpression of *CKS2* in PDAC compared with normal tissues has also been reported in the Oncomine database (Fig. 4), and data from the cbioportal database showed that *CKS2* is amplified in many types of cancer (Fig. 5a). *CKS2* expression levels positively correlate with copy number in 15 tumor cell lines (Fig. 5b).

**Table 2:** Fold differences in expression of *CKS2*, *ITGA3*, *BIRC5*, *RALA* and *ITGA6* between pancreatic cancer tissue and normal tissue in six GEO microarray datasets

Gene	Fold difference^a^						Average fold difference
	GSE71989	GSE15471	GSE16515	GSE32676	GSE41368	GSE28735	
*CKS2*	6.61	3.18	4.32		5.17		4.82
*ITGA3*	2.32	2.51	3.01	3.49	4.74	7.95	4.00
*BIRC5*	2.42		2.37	2.11			2.30
*RALA*	2.12	2.02	2.44	2.03			2.15
*ITGA6*	2.01			2.20			2.11

^a^Positive differences indicate up-regulation in pancreatic tumor tissue relative to non-tumor tissue. Fold differences of at least 2 were regarded as significant, so differences below 2 were left blank in this table

### CKS2 expression negatively correlates with DNA methylation

Tumor cells show dysregulation of DNA methylation levels and patterns, leading to significant changes in the overall level of DNA methylation in tumors [11]. Data in The Cancer Genome Atlas for 149 PDAC tissue samples show that methylation levels at the *CKS2* locus negatively correlate with *CKS2* expression (*r *= − 0.368, *P *< 0.001; Fig. 5c).

### CKS2 promotes PDAC cell proliferation

**Fig. 6:**
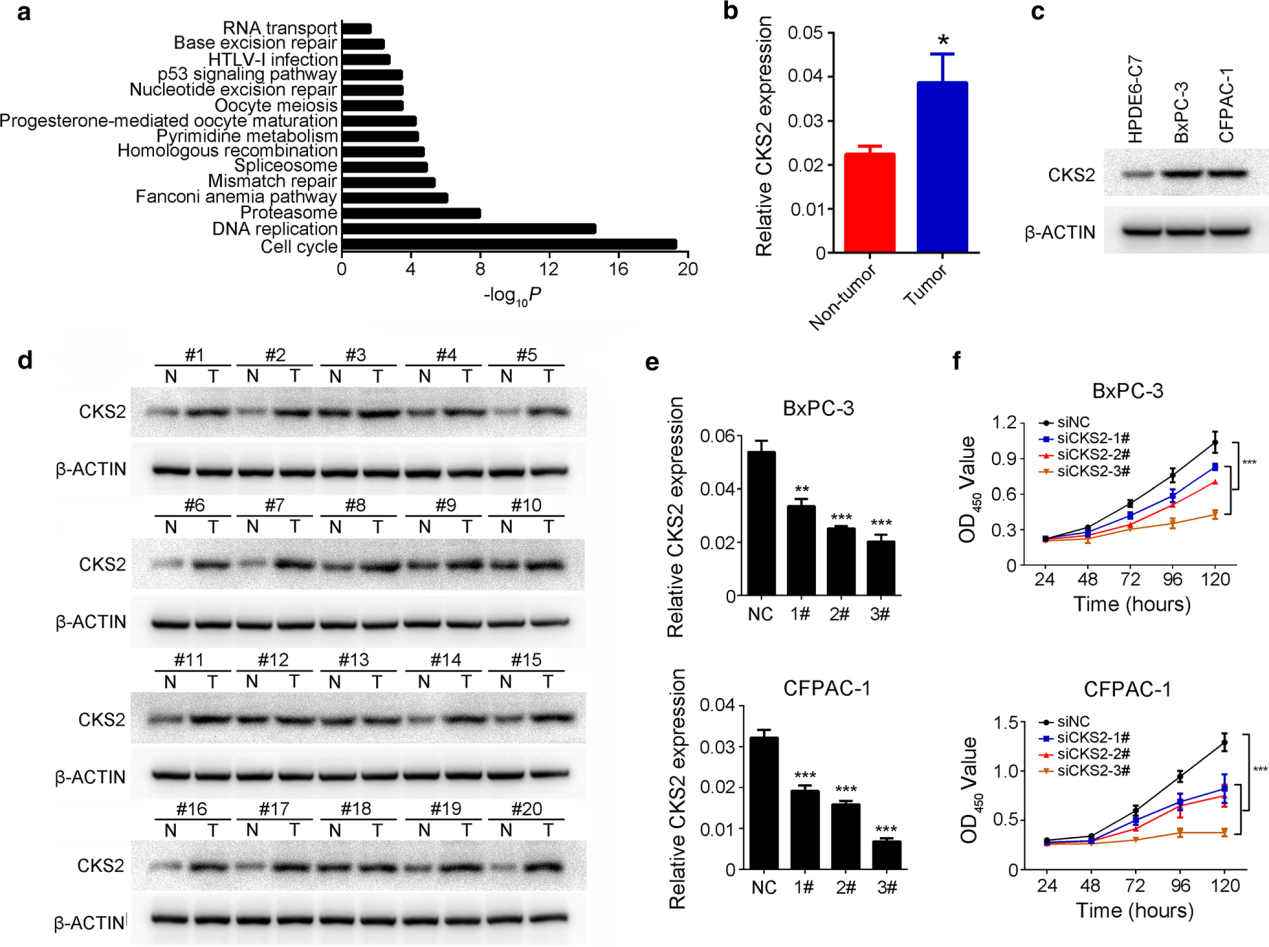
*CKS2* promotes pancreatic cancer cell proliferation. **a** Significant pathways enriched in genes whose expression profile matches that of *CKS2,* based on DAVID software. **b** *CKS2* mRNA levels in pancreatic cancer tissues and paired normal tissues from 102 patients with PDAC at our hospital, based on real-time PCR analysis. Results are mean ± SEM. *P < 0.05. **c** Western blotting of *CKS2* in PDAC cell lines BxPC-3 and CFPAC-1, as well as in non-cancer pancreatic duct epithelial cell line HPDE6-C7. **d** Western blotting of *CKS2* in PDAC (T) and paired normal tissues (N). Representative results of 20 pairs of clinical specimens are shown. **e** *CKS2* knockdown in BxPC-3 and CFPAC-1 cells. Levels of mRNAs were determined by qRT-PCR. Results are mean ± SEM from three independent experiments. **f** *CKS2* knockdown substantially reduced proliferation of BxPC-3 and CFPAC-1 cells, as determined using the CCK-8 assay. Results are mean ± SEM from three experiments, each conducted with six replicates of each sample. ***P < 0.001 vs. control

To characterize the potential functional role of *CKS2*, we analyzed genes whose expression parallels that of *CKS2* based on RNA sequencing data from PDAC tissues in The Cancer Genome Atlas. A total of 325 genes co-expressed with *CKS2* were identified (absolute *r *> 0.5; Additional file 6: Table S6), and these genes were enriched in the cell cycle pathway (Fig. 6a). Analysis of tumor and non-tumor tissues from our cohort of 102 patients with PDAC showed that *CKS2* mRNA levels were higher in tumor tissues (Fig. 6b). *CKS2* protein levels were higher in PDAC cell lines BxPC-3 and CFPAC-1 than in immortalized non-cancer pancreatic duct epithelial cell line HPDE6-C7 (Fig. 6c). Consistent with these results, *CKS2* protein levels were higher in 20 samples of pancreatic cancer tissue than in the corresponding samples of adjacent normal tissue (Fig. 6d). Knockdown of *CKS2* expression in the PDAC cell lines BxPC-3 and CFPAC-1 substantially suppressed proliferation (Fig. 6e, f).

## Discussion

In this study, we analyzed PDAC-associated genes and pathways in six frequently cited, published pancreatic cancer microarray datasets in the GEO database: GSE71989 [12, 13], GSE15471 [14], GSE16515 [15, 16], GSE32676 [17, 18], GSE41368 [19], and GSE28735 [18, 20]. We investigated differences in gene expression between pancreatic cancer tissues and normal tissues. Significant pathways enriched for differentially expressed genes were assembled into a pathway network, and core pathways showing high centrality degree in this network were chosen for further study. The pathway in cancer (KEGGID: 05200) was the top core pathway, and one gene in this pathway, *CKS2*, has been shown to be overexpressed in PDAC relative to normal tissue. *CKS2* expression correlates positively with copy number and negatively with DNA methylation. *CKS2* overexpression may be associated with shorter survival of patients with PDAC, probably due to *CKS2* function in promoting proliferation of pancreatic cancer cells.

We used essentially all samples from these six GEO datasets to identify genes differentially expressed between PDAC tissue and normal tissue, although we excluded some samples to avoid the batch effect in previous studies [21]. This also allowed us to pool the results from each dataset to perform integrative analysis. As part of this analysis, we created pathway networks [22], which were introduced to mine GEO data. Molecules in different signaling pathways interact with one another, so the pathways in a cell can be connected into a system. This is, to the best of our knowledge, the first analysis of PDAC-associated genes at the level of pathway networks. We obtained different pathway networks from the six GEO datasets, reflecting differences in samples, testing platform and other reasons (Additional file 7: Figure S1). If a pathway is important for pancreatic cancer development or progression, it should appear consistently across microarray experiments. We found 32 significant pathways common to all six GEO datasets, and within those, 13 core pathways, which are particularly likely to be important in the development and progression of pancreatic cancer. Of these core pathways, the pathway in cancer (KEGG ID: 05200) showed the highest degree centrality, suggesting that it is the most important pathway in pancreatic cancer. The pathway in cancer is an integrated signaling pathway that includes RAS-mediated pathways [23]. KRAS is a well-known driver gene for pancreatic cancer, and mutation of this gene occurs in about 90% of patients [24]. This suggests that our pathway network analysis can give reliable results.

We then analyzed whether all differentially expressed genes in the pathway in cancer across six GEO datasets are associated with PDAC survival, based on data in The Cancer Genome Atlas and International Cancer Genome Consortium databases. In both databases, expression of *BIRC5*, *CKS2*, *ITGA3*, *ITGA6* and *RALA* were significantly associated with survival time. We did not identify any expression quantitative trait loci associated with differential expression of these five genes in pancreatic tissues, based on data in the GTEx database [25]. This result may reflect the limited sample size. Of the five differentially expressed genes, *CKS2* showed the greatest dysregulation in PDAC, which may be due to changes in copy number or/and DNA methylation (Fig. 5b, c).

*CKS2*, a gene encoding CDC28 protein kinase regulatory subunit, is located in chromosomal region 9q22.2. Previous studies have reported aberrant expression of this gene in cancers of the bladder [26], stomach [27], prostate [28], esophagus [29] and breast [30]. Our database mining showed that many types of tumor overexpress *CKS2* (Additional file 8: Figure S2 and Additional file 9: S3). Although *CKS2* has been shown to play an important role in many types of cancer, its function in PDAC has not been reported. Our database mining showed that the genes associated with *CKS2* are involved mainly in cell proliferation processes, such as the cell cycle and DNA replication. This implies that *CKS2* knockdown may inhibit PDAC cell proliferation, which we confirmed directly in cultures of PDAC cell lines. These results are consistent with those obtained with other types of cancer.

*BIRC5* is a member of the inhibitor of apoptosis gene family, which is overexpressed in many types of tumors [31, 32]. *BIRC5* expression can be regulated by STAT3 [33], and it can be suppressed by curcumin, which induces apoptosis and ultimately inhibits PDAC cell proliferation [34]. *ITGA3* and *ITGA6* are two members of the integrin alpha chain family of proteins. *ITGA3* may promote proliferation or invasion in various types of cancer [35–37]. *ITGA6* expression has been correlated with expression of migration-related genes [38], and it can promote the epithelial-mesenchymal transition [39] and tumor invasion [40]. *ITGA6* also plays a role in tumorigenesis [41]. *ITGA3* and *ITGA6,* both members of the integrin signaling pathway, are overexpressed in pancreatic cancer [42]. *RALA* may participate in pancreatic cancer initiation [43]. The details of how these genes participate in pancreatic cancer development and progression require further investigation.

## Conclusions

Integrative analysis of six GEO microarray datasets of pancreatic cancer suggests that the pathway in cancer (KEGG ID: 05200) is an essential pathway in the development and progression of PDAC. High *CKS2* expression is significantly associated with shorter survival time in patients with PDAC, which may reflect a function of *CKS2* in promoting PDAC cell proliferation. These results demonstrate the potential of integrative data-mining from the GEO database in order to gain further insights into PDAC and other diseases.

## Additional files

**Additional file 1: Table S1.** Characteristics of patients with pancreatic cancer in this study.

**Additional file 2: Table S2.** Short interfering RNA (siRNA) sequences used in this study.

**Additional file 3: Table S3.** Primers used for real-time PCR in this study.

**Additional file 4: Table S4.** Pancreatic cancer microarray datasets included in the study.

**Additional file 5: Table S5.** Hazard ratio (HR) of death for genes in the pathway in cancer that are differentially expressed in pancreatic cancer, based on data in two databases.

**Additional file 6: Table S6.** Genes associated with *CKS2* expression level in pancreatic cancer tissues, based on data in The Cancer Genome Atlas.

**Additional file 7: Figure S1.** Pathway networks of all GEO datasets. Pathway networks for the datasets GSE15471 **(A)**, GSE16515 **(B)**, GSE32676 **(C)**, GSE71989 **(D)**, GSE28735 **(E)** and GSE42368 **(F)** were drawn using the GCBI online tool as described in Methods. Yellow dots indicate involvement of up- and down-regulated signaling pathway genes; red dots, up-regulated signaling pathway genes; and blue dots, down-regulated signaling pathway genes. The arrow points from the upstream toward the downstream signaling pathway.

**Additional file 8: Figure S2.** *CKS2* expression in different types of cancer cell lines, based on data from the CCLE database (https://portals.broadinstitute.org/ccle/home).

**Additional file 9: Figure S3.** *CKS2* expression across multiple types of cancer and corresponding normal tissues. The numbers of studies reporting up- or down-regulation is indicated, respectively, in red or blue boxes. Color intensity reflects the best gene rank percentile for analyses within the box. The following settings were used for the analysis: *P*=0.05, fold change=all, gene rank=all, and data type=mRNA (http://www.oncomine.org).

## Abbreviations

CCLE: cancer cell line encyclopedia
FBS: fetal bovine serum
GEO: gene expression omnibus
ICGC: International Cancer Genome Consortium
PDAC: pancreatic duct adenocarcinoma
siRNA: short interfering RNA
TCGA: The Cancer Genome Atlas
