# Supplementary material for: Integrative analysis of gene expression profiles reveals specific signaling pathways associated with pancreatic duct adenocarcinoma
Source: Cancer Commun (Lond). 2018 Apr 27;38:13. doi: 10.1186/s40880-018-0289-9 (PMC5993144; doi:10.1186/s40880-018-0289-9)
Supplement: Supplementary file 1 — Additional file 1: Table S1. Characteristics of patients with pancreatic cancer in this study. [file 40880_2018_289_MOESM1_ESM.docx]

Additional file 1: Table S1. Characteristics of patients with pancreatic cancer in this study

| Variable | Alive (N=38) | Deceased (N=64) | *P* |
| --- | --- | --- | --- |
| Age, mean (SD) | 59.26 (2.16) | 60.50 (1.52) | 0.640 |
|  |  |  |  |
| Gender, *N* (%) |  |  | 0.506 |
| Male | 20 (52.6) | 38 (59.4) |  |
| Female | 18 (47.4) | 26 (40.6) |  |
|  |  |  |  |
| Differentiation, *N* (%) |  |  | 0.616 |
| Well | 7 (18.4) | 17 (26.6) |  |
| Moderate | 24 (63.2) | 35 (54.7) |  |
| Poor | 7 (18.4) | 12 (18.8) |  |
|  |  |  |  |
| Lymph node status, *N* (%) |  |  | **0.028** |
| Positive | 14 (36.8) | 38 (59.4) |  |
| Negative | 24 (63.2) | 26 (40.6) |  |
|  |  |  |  |
| Vascular invasion, *N* (%) |  |  | 0.156 |
| Yes | 7 (18.4) | 20 (31.2) |  |
| No | 31 (81.6) | 44 (68.8) |  |
|  |  |  |  |
| Neural invasion, *N* (%) |  |  | 0.254 |
| Yes | 14 (36.8) | 31 (48.4) |  |
| No | 24 (63.2) | 33 (51.6) |  |
|  |  |  |  |
| Tumor stage, *N* (%) |  |  | **0.011** |
| I | 3 (7.9) | 4 (6.3) |  |
| II | 23 (60.5) | 55 (85.9) |  |
| III | 2 (5.3) | 2 (3.1) |  |
| IV | 10 (26.3) | 3 (4.7) |  |
|  |  |  |  |
| Recurrence, *N* (%) |  |  | 0.146 |
| Yes | 4 (10.5) | 14 (21.9) |  |
| No | 34 (89.5) | 50 (78.1) |  |
|  |  |  |  |
| Metastatic, *N* (%) |  |  | 0.256 |
| Yes | 9 (23.7) | 22 (34.4) |  |
| No | 29 (76.3) | 42 (65.6) |  |
|  |  |  |  |
| Smoking status, *N* (%) |  |  | 0.802 |
| Ever | 17 (44.7) | 27 (42.2) |  |
| Never | 21 (55.3) | 37 (57.8) |  |
|  |  |  |  |
| Drinking status, *N* (%) |  |  | 0.206 |
| Ever | 5 (13.2) | 15 (23.4) |  |
| Never | 33 (86.8) | 49 (76.6) |  |
|  |  |  |  |
| Treatment, *N* (%) |  |  | 0.879 |
| Surgery only | 19 (50.0) | 33 (51.6) |  |
| Surgery + Chemotherapy | 19 (50.0) | 31 (48.4) |  |
