# Supplementary material for: Integrative analysis of gene expression profiles reveals specific signaling pathways associated with pancreatic duct adenocarcinoma
Source: Cancer Commun (Lond). 2018 Apr 27;38:13. doi: 10.1186/s40880-018-0289-9 (PMC5993144; doi:10.1186/s40880-018-0289-9)
Supplement: Supplementary file 2 — Additional file 2: Table S2. Short interfering RNA (siRNA) sequences used in this study. [file 40880_2018_289_MOESM2_ESM.docx]

Additional file 2: Table S2. Short interfering RNA (siRNA) sequences used in this study

| siRNA | Sequence (5’-3’) |
| --- | --- |
| siNC | TTC TCC GAA CGT GTC ACG TTT |
| siCKS2-1# | GTA CCG GCA TGT TAT GTT ATT |
| siCKS2-2# | GCT GGG TTC ATT ACA TGA TTT |
| siCKS2-3# | TCT CTT TAG ACG ACC TCT TTT |
