# Supplementary material for: Integrative analysis of gene expression profiles reveals specific signaling pathways associated with pancreatic duct adenocarcinoma
Source: Cancer Commun (Lond). 2018 Apr 27;38:13. doi: 10.1186/s40880-018-0289-9 (PMC5993144; doi:10.1186/s40880-018-0289-9)
Supplement: Supplementary file 3 — Additional file 3: Table S3. Primers used for real-time PCR in this study. [file 40880_2018_289_MOESM3_ESM.docx]

Additional file 3: Table S3. Primers used for real-time PCR in this study

| Primer | Sequence(5’-3’) |
| --- | --- |
| CKS2-forward | CAC TAC GAG TAC CGG CAT GTT |
| CKS2-reverse | CAT GTA ATG AAC CCA GCC TAG A |
| GAPDH-forward | TTG GCC AGG GGT GCT AAG |
| GAPDH-reverse | AGC CAA AAG GGT CAT CAT CTC |

GAPDH, glyceraldehyde 3-phosphate dehydrogenase
