# Supplementary material for: Integrative analysis of gene expression profiles reveals specific signaling pathways associated with pancreatic duct adenocarcinoma
Source: Cancer Commun (Lond). 2018 Apr 27;38:13. doi: 10.1186/s40880-018-0289-9 (PMC5993144; doi:10.1186/s40880-018-0289-9)
Supplement: Supplementary file 4 — Additional file 4: Table S4. Pancreatic cancer microarray datasets included in the study. [file 40880_2018_289_MOESM4_ESM.docx]

Additional file 4: Table S4. Pancreatic cancer microarray datasets included in the study

| Platform | Dataset | GEO accession no. | Sample type | Number of samples | |
| --- | --- | --- | --- | --- | --- |
|  |  |  |  | Normal | Tumor |
| GPL570 | 1 | GSE15471 | Paired tissues | 39 | 39 |
|  | 2 | GSE16515 | Non-paired tissues | 16 | 36 |
|  | 3 | GSE32676 | Non-paired tissues | 7 | 25 |
|  | 4 | GSE71989 | Non-paired tissues | 8 | 14 |
| GPL6244 | 5 | GSE28735 | Paired tissues | 45 | 45 |
|  | 6 | GSE41368 | Non-paired tissues | 6 | 6 |
| Total |  |  |  | 121 | 165 |
