# Supplementary material for: Integrative analysis of gene expression profiles reveals specific signaling pathways associated with pancreatic duct adenocarcinoma
Source: Cancer Commun (Lond). 2018 Apr 27;38:13. doi: 10.1186/s40880-018-0289-9 (PMC5993144; doi:10.1186/s40880-018-0289-9)
Supplement: Supplementary file 5 — Additional file 5: Table S5. Hazard ratio (HR) of death for genes in the pathway in cancer that are differentially expressed in pancreatic cancer, based on data in two databases. [file 40880_2018_289_MOESM5_ESM.docx]

Additional file 5: Table S5. Hazard ratio (HR) of death for genes in the pathway in cancer that are differentially expressed in pancreatic cancer, based on data in two databases

| Gene | The Cancer Genome Atlas | | |  | International Cancer Genome Consortium | | |
| --- | --- | --- | --- | --- | --- | --- | --- |
|  | HR | 95 % Cl | *P* |  | HR | 95 % Cl | *P* |
| *AKT3* | 1.16 | 0.77‒1.74 | 0.4839 |  | 1.29 | 0.88‒1.89 | 0.1876 |
| *APC* | 1.15 | 0.76‒1.67 | 0.5057 |  | 1.33 | 0.91‒1.95 | 0.1358 |
| *BAX* | 1.36 | 0.90‒2.07 | 0.1467 |  | 0.98 | 0.67‒1.44 | 0.9332 |
| *BCL2* | 1.21 | 0.80‒1.82 | 0.3713 |  | 1.38 | 0.95‒2.02 | 0.0922 |
| *BCL2L1* | 1.89 | 1.24‒2.88 | 0.0033 |  | 0.80 | 0.54‒1.17 | 0.2444 |
| *BCR* | 1.08 | 0.72‒1.64 | 0.7035 |  | 1.18 | 0.81‒1.73 | 0.3830 |
| *BID* | 1.29 | 0.85‒1.94 | 0.2287 |  | 0.95 | 0.65‒1.39 | 0.8012 |
| *BIRC3* | 1.46 | 0.97‒2.21 | 0.0731 |  | 1.61 | 1.10‒2.36 | 0.0128 |
| ***BIRC5*** | **1.63** | **1.08‒2.48** | **0.0214** |  | **2.14** | **1.46‒3.15** | **0.0001** |
| *BMP4* | 1.67 | 1.10‒2.53 | 0.0162 |  | 1.00 | 0.69‒1.46 | 0.9945 |
| *CASP3* | 1.77 | 1.16‒2.69 | 0.0080 |  | 1.26 | 0.86‒1.84 | 0.2324 |
| *CASP8* | 1.44 | 0.94‒2.19 | 0.0900 |  | 1.18 | 0.80‒1.72 | 0.3991 |
| *CBL* | 1.11 | 0.73‒1.67 | 0.6276 |  | 1.00 | 0.68‒1.45 | 0.9796 |
| *CBLB* | 1.26 | 0.83‒1.90 | 0.2748 |  | 1.16 | 0.80‒1.70 | 0.4350 |
| *CBLC* | 1.39 | 0.92‒2.10 | 0.1213 |  | 1.30 | 0.89‒1.90 | 0.1689 |
| *CCND1* | 1.68 | 1.11‒2.55 | 0.0151 |  | 1.31 | 0.90‒1.90 | 0.1633 |
| *CDC42* | 1.19 | 0.79‒1.79 | 0.4121 |  | 1.36 | 0.93‒1.99 | 0.1085 |
| *CDH1* | 1.09 | 0.72‒1.64 | 0.6924 |  | 0.86 | 0.59‒1.26 | 0.4472 |
| *CDK6* | 2.22 | 1.44‒3.41 | 0.0003 |  | 0.93 | 0.64‒1.36 | 0.7065 |
| *CDKN2B* | 1.39 | 0.92‒2.09 | 0.1219 |  | 0.92 | 0.63‒1.34 | 0.6482 |
| *CKS1B* | 1.66 | 1.09‒2.52 | 0.0183 |  | 0.89 | 0.61‒1.29 | 0.5341 |
| ***CKS2*** | **1.74** | **1.14‒2.64** | **0.0100** |  | **1.52** | **1.03‒2.22** | **0.0328** |
| *COL4A1* | 1.17 | 0.77‒1.78 | 0.4524 |  | 1.17 | 0.80‒1.71 | 0.4089 |
| *COL4A2* | 1.02 | 0.68‒1.54 | 0.9133 |  | 0.99 | 0.68‒1.45 | 0.9706 |
| *COL4A3* | 0.78 | 0.52‒1.18 | 0.2443 |  | 1.27 | 0.86‒1.87 | 0.2233 |
| *COL4A5* | 0.96 | 0.64‒1.44 | 0.8371 |  | 1.49 | 1.02‒2.17 | 0.0396 |
| *CSF1R* | 1.13 | 0.75‒1.71 | 0.5510 |  | 1.33 | 0.91‒1.94 | 0.1367 |
| *CSF2RA* | 1.13 | 0.75‒1.72 | 0.5510 |  | 1.36 | 0.93‒1.99 | 0.1102 |
| *CSF3R* | 0.95 | 0.63‒1.44 | 0.8102 |  | 1.01 | 0.69‒1.47 | 0.9719 |
| *CTNNA1* | 1.71 | 1.12‒2.61 | 0.0122 |  | 1.32 | 0.91‒1.93 | 0.1453 |
| *CTNNB1* | 1.17 | 0.77‒1.77 | 0.4645 |  | 1.14 | 0.78‒1.66 | 0.4888 |
| *CYCS* | 1.08 | 0.71‒1.63 | 0.7182 |  | 1.21 | 0.83‒1.76 | 0.3273 |
| *DVL3* | 0.98 | 0.65‒1.48 | 0.9202 |  | 1.02 | 0.71‒1.52 | 0.9329 |
| *E2F3* | 1.47 | 0.97‒2.23 | 0.0696 |  | 0.99 | 0.68‒1.43 | 0.9372 |
| *EGF* | 1.19 | 0.79‒1.80 | 0.4000 |  | 1.18 | 0.81‒1.72 | 0.3861 |
| *EGLN3* | 1.38 | 0.91‒2.08 | 0.1300 |  | 1.03 | 0.71‒1.51 | 0.8637 |
| *EP300* | 1.26 | 0.83‒1.90 | 0.2767 |  | 1.08 | 0.74‒1.58 | 0.6884 |
| *ERBB2* | 1.47 | 0.97‒2.23 | 0.0668 |  | 1.51 | 1.03‒2.22 | 0.0325 |
| *ETS1* | 1.29 | 0.86‒1.96 | 0.2189 |  | 1.31 | 0.89‒1.91 | 0.1662 |
| *FAS* | 1.90 | 1.25‒2.89 | 0.0025 |  | 1.22 | 0.83‒1.78 | 0.3063 |
| *FGF12* | Not found | | |  | 1.23 | 0.85‒1.80 | 0.2575 |
| *FGF6* | Not found | | |  | 1.08 | 0.74‒1.57 | 0.6997 |
| *FGF7* | 0.93 | 0.62‒1.40 | 0.7237 |  | 1.18 | 0.81‒1.72 | 0.3984 |
| *FGFR1* | 1.49 | 0.98‒2.25 | 0.0602 |  | 1.12 | 0.77‒1.63 | 0.5560 |
| *FGFR2* | 1.07 | 0.71‒1.61 | 0.7622 |  | 1.14 | 0.78‒1.67 | 0.5052 |
| *FLT3* | 1.03 | 0.68‒1.55 | 0.8926 |  | 0.95 | 0.65‒1.38 | 0.7744 |
| *FN1* | 1.37 | 0.91‒2.08 | 0.1400 |  | 1.25 | 0.85‒1.82 | 0.2546 |
| *FOS* | 1.14 | 0.76‒1.72 | 0.5304 |  | 1.05 | 0.72‒1.54 | 0.7852 |
| *FZD1* | 1.28 | 0.85‒1.93 | 0.2426 |  | 0.86 | 0.59‒1.26 | 0.4486 |
| *FZD2* | 1.47 | 0.98‒2.23 | 0.0656 |  | 1.31 | 0.90‒1.91 | 0.1620 |
| *FZD6* | 1.91 | 1.25‒2.93 | 0.0028 |  | 1.35 | 0.93‒1.97 | 0.1151 |
| *FZD7* | 1.09 | 0.73‒1.65 | 0.6679 |  | 1.13 | 0.77‒1.64 | 0.5379 |
| *GLI2* | 1.21 | 0.80‒1.82 | 0.3659 |  | 1.34 | 0.91‒1.95 | 0.1323 |
| *GLI3* | 1.29 | 0.85‒1.94 | 0.2298 |  | 1.44 | 0.98‒2.11 | 0.0584 |
| *HDAC1* | 1.21 | 0.80‒1.82 | 0.3735 |  | 1.17 | 0.80‒1.71 | 0.4046 |
| *HGF* | 0.95 | 0.63‒1.43 | 0.7951 |  | 1.11 | 0.75‒1.62 | 0.6074 |
| *HHIP* | 1.50 | 0.99‒2.26 | 0.0562 |  | 1.16 | 0.80‒1.69 | 0.4349 |
| *HIF1A* | 1.10 | 0.73‒1.67 | 0.6398 |  | 1.35 | 0.92‒1.98 | 0.1189 |
| *HSP90AA1* | 1.32 | 0.87‒2.00 | 0.1943 |  | 1.11 | 0.76‒1.61 | 0.5985 |
| *IGF1* | 0.95 | 0.63‒1.44 | 0.8145 |  | 1.03 | 0.71‒1.50 | 0.8768 |
| *IKBKB* | 0.96 | 0.64‒1.45 | 0.8592 |  | 1.13 | 0.78‒1.66 | 0.5144 |
| *IL6* | 1.31 | 0.87‒1.98 | 0.1931 |  | 1.00 | 0.68‒1.46 | 0.9930 |
| *IL8* | 1.11 | 0.73‒1.67 | 0.6300 |  | 1.15 | 0.79‒1.69 | 0.4581 |
| *ITGA2* | 1.75 | 1.15‒2.68 | 0.0100 |  | 1.29 | 0.88‒1.89 | 0.1900 |
| *ITGA2B* | Not found | | |  | 0.89 | 0.61‒1.30 | 0.5603 |
| ***ITGA3*** | **1.73** | **1.13‒2.65** | **0.0100** |  | **1.59** | **1.08‒2.34** | **0.0200** |
| ***ITGA6*** | **1.68** | **1.10‒2.55** | **0.0152** |  | **1.61** | **1.10‒2.35** | **0.0141** |
| *ITGAV* | 1.47 | 0.97‒2.23 | 0.0697 |  | 0.96 | 0.66‒1.41 | 0.8395 |
| *ITGB1* | 1.37 | 0.90‒2.07 | 0.1406 |  | 1.27 | 0.87‒1.85 | 0.2168 |
| *JUP* | 1.44 | 0.95‒2.17 | 0.0855 |  | 1.05 | 0.72‒1.54 | 0.7818 |
| *KIT* | 1.08 | 0.72‒1.63 | 0.7117 |  | 1.04 | 0.71‒1.51 | 0.8512 |
| *KITLG* | 1.72 | 1.13‒2.61 | 0.0113 |  | 1.06 | 0.73‒1.54 | 0.7668 |
| *KLK3* | Not found | | |  | 1.26 | 0.86‒1.83 | 0.2358 |
| *LAMA2* | 1.19 | 0.79‒1.8 | 0.3977 |  | 1.27 | 0.87‒1.85 | 0.2218 |
| *LAMA3* | 2.08 | 1.36‒3.18 | 0.0006 |  | 1.17 | 0.80‒1.71 | 0.417 |
| *LAMA4* | 1.16 | 0.77‒1.75 | 0.4722 |  | 1.37 | 0.93‒1.99 | 0.1062 |
| *LAMB1* | 1.22 | 0.81‒1.85 | 0.3384 |  | 1.14 | 0.78‒1.67 | 0.4947 |
| *LAMB2* | 1.11 | 0.74‒1.67 | 0.6228 |  | 1.22 | 0.83‒1.79 | 0.3056 |
| *LAMB3* | 1.78 | 1.17‒2.70 | 0.0100 |  | 1.03 | 0.71‒1.51 | 0.8700 |
| *LAMC1* | 1.10 | 0.73‒1.66 | 0.6416 |  | 1.50 | 1.03‒2.20 | 0.0347 |
| *LAMC2* | 1.33 | 0.88‒2.02 | 0.1800 |  | 1.58 | 1.08‒2.31 | 0.0185 |
| *LEF1* | 1.33 | 0.88‒2.01 | 0.1700 |  | 1.20 | 0.82‒1.75 | 0.3390 |
| *MAPK1* | 1.44 | 0.95‒2.18 | 0.0829 |  | 1.29 | 0.88‒1.88 | 0.1860 |
| *MAPK9* | 1.13 | 0.75‒1.70 | 0.5586 |  | 1.27 | 0.87‒1.85 | 0.2151 |
| *MECOM* | 1.62 | 1.06‒2.46 | 0.0249 |  | 1.19 | 0.81‒1.74 | 0.3790 |
| *MET* | 2.42 | 1.56‒3.76 | 0.0000 |  | 1.28 | 0.88‒1.87 | 0.2000 |
| *MITF* | 1.28 | 0.85‒1.93 | 0.2456 |  | 1.10 | 0.75‒1.62 | 0.6160 |
| *MMP1* | 1.58 | 1.04‒2.40 | 0.0300 |  | 1.09 | 0.74‒1.58 | 0.6700 |
| *MMP2* | 1.04 | 0.69‒1.57 | 0.8600 |  | 1.32 | 0.90‒1.93 | 0.1525 |
| *MMP9* | 1.38 | 0.91‒2.09 | 0.1300 |  | 1.14 | 0.78‒1.66 | 0.4889 |
| *MYC* | 1.35 | 0.90‒2.05 | 0.1506 |  | 1.28 | 0.87‒1.86 | 0.2059 |
| *NFKB1* | 1.17 | 0.77‒1.77 | 0.4623 |  | 1.16 | 0.80‒1.69 | 0.4412 |
| *NFKBIA* | 0.93 | 0.62‒1.40 | 0.7323 |  | 1.02 | 0.70‒1.48 | 0.9349 |
| *PDGFRA* | 0.97 | 1.65‒1.47 | 0.8982 |  | 1.00 | 0.69‒1.47 | 0.9821 |
| *PDGFRB* | 0.99 | 0.66‒1.50 | 0.9706 |  | 1.04 | 0.71‒1.52 | 0.8323 |
| *PIK3CA* | 1.37 | 0.91‒2.07 | 0.1362 |  | 0.84 | 0.58‒1.23 | 0.3803 |
| *PIK3CD* | 1.00 | 0.66‒1.51 | 0.9980 |  | 1.03 | 0.71‒1.50 | 0.8764 |
| *PIK3CG* | 1.18 | 0.78‒1.80 | 0.4325 |  | 1.12 | 0.77‒1.63 | 0.5633 |
| *PIK3R1* | 1.13 | 0.75‒1.71 | 0.5508 |  | 0.99 | 0.68‒1.45 | 0.9542 |
| *PIK3R5* | 0.81 | 0.53‒1.22 | 0.3146 |  | 1.25 | 0.85‒1.82 | 0.2542 |
| *PLD1* | 1.02 | 0.68‒1.54 | 0.9125 |  | 1.28 | 0.88‒1.86 | 0.1989 |
| *PPARD* | 1.15 | 0.76‒1.75 | 0.5057 |  | 1.21 | 0.83‒1.77 | 0.3276 |
| *PPARG* | 1.47 | 0.97‒2.22 | 0.0700 |  | 1.02 | 0.70‒1.49 | 0.9090 |
| *PRKCA* | 0.98 | 0.65‒1.48 | 0.9187 |  | 1.28 | 0.87‒1.87 | 0.2050 |
| *PRKCB* | 1.21 | 0.80‒1.83 | 0.3633 |  | 1.06 | 0.73‒1.55 | 0.7450 |
| *PTGS2* | 1.22 | 0.81‒1.84 | 0.3436 |  | 1.21 | 0.83‒1.76 | 0.3333 |
| *PTK2* | 1.40 | 0.92‒2.12 | 0.1162 |  | 1.49 | 1.01‒2.18 | 0.0415 |
| *RAC1* | 1.57 | 1.03‒2.38 | 0.0352 |  | 0.98 | 0.67‒1.43 | 0.9094 |
| *RAC2* | 1.05 | 0.69‒1.58 | 0.8316 |  | 1.13 | 0.78‒1.65 | 0.5105 |
| ***RALA*** | **1.81** | **1.19‒2.76** | **0.0060** |  | **1.61** | **1.09‒2.37** | **0.0158** |
| *RALBP1* | 1.70 | 1.12‒2.59 | 0.0127 |  | 1.19 | 0.82‒1.73 | 0.3671 |
| *RARA* | 1.38 | 0.91‒2.10 | 0.1272 |  | 1.41 | 0.96‒2.06 | 0.0754 |
| *RARB* | 1.25 | 0.82‒1.89 | 0.2986 |  | 1.52 | 1.04‒2.23 | 0.0304 |
| *RB1* | 1.40 | 0.93‒2.12 | 0.1067 |  | 0.89 | 0.60‒1.31 | 0.5503 |
| *RBX1* | 1.34 | 0.89‒2.03 | 0.1587 |  | 1.05 | 0.72‒1.53 | 0.8108 |
| *RUNX1* | 1.04 | 0.69‒1.56 | 0.8666 |  | 1.11 | 0.76‒1.63 | 0.5893 |
| *RUNX1T1* | 1.02 | 0.68‒1.54 | 0.9169 |  | 1.13 | 0.78‒1.65 | 0.5216 |
| *SKP2* | 1.38 | 0.91‒2.08 | 0.1310 |  | 1.54 | 1.05‒2.25 | 0.0254 |
| *SLC2A1* | 1.46 | 0.96‒2.22 | 0.0700 |  | 1.21 | 0.83‒1.76 | 0.3295 |
| *SOS1* | 1.27 | 0.84‒1.92 | 0.2644 |  | 1.06 | 0.73‒1.54 | 0.7709 |
| *STAT1* | 1.65 | 1.05‒2.50 | 0.0179 |  | 1.04 | 0.71‒1.52 | 0.8408 |
| *STAT3* | 1.04 | 0.69‒1.56 | 0.8602 |  | 1.23 | 0.84‒1.79 | 0.2842 |
| *STK4* | 1.00 | 0.66‒1.52 | 0.9845 |  | 0.96 | 0.66‒1.40 | 0.8450 |
| *SUFU* | 1.40 | 0.92‒2.14 | 0.1140 |  | 1.01 | 0.69‒1.48 | 0.9583 |
| *TCF7L2* | 1.49 | 0.98‒2.26 | 0.0595 |  | 0.89 | 0.61‒1.30 | 0.5362 |
| *TGFA* | 1.79 | 1.17‒2.73 | 0.0072 |  | 1.23 | 0.84‒1.79 | 0.2882 |
| *TGFB1* | 1.12 | 0.74‒1.69 | 0.6049 |  | 0.87 | 0.59‒1.27 | 0.4690 |
| *TGFB2* | 1.48 | 0.97‒2.27 | 0.0678 |  | 1.20 | 0.83‒1.76 | 0.3327 |
| *TGFB3* | 0.98 | 0.65‒1.48 | 0.9216 |  | 1.01 | 0.69‒1.47 | 0.9738 |
| *TGFBR1* | 1.32 | 0.87‒1.99 | 0.1879 |  | 1.21 | 0.83‒1.77 | 0.3308 |
| *TPR* | 1.38 | 0.91‒2.10 | 0.1242 |  | 1.12 | 0.77‒1.64 | 0.5410 |
| *VEGFA* | 0.88 | 0.58‒1.33 | 0.5471 |  | 1.35 | 0.92‒1.97 | 0.1234 |
| *VEGFC* | 0.88 | 0.58‒1.33 | 0.5450 |  | 1.44 | 0.98‒2.11 | 0.0589 |
| *WNT2* | 1.59 | 1.05‒2.42 | 0.0286 |  | 1.16 | 0.79‒1.68 | 0.4507 |
| *WNT5A* | 1.52 | 1.00‒2.31 | 0.0494 |  | 1.12 | 0.77‒1.64 | 0.5519 |
| *WNT6* | Not found | | |  | 1.16 | 0.79‒1.69 | 0.4489 |
| *ZBTB16* | 0.97 | 0.64‒1.46 | 0.8875 |  | 1.23 | 0.84‒1.79 | 0.2895 |
