# Supplementary material for: Integrative analysis of gene expression profiles reveals specific signaling pathways associated with pancreatic duct adenocarcinoma
Source: Cancer Commun (Lond). 2018 Apr 27;38:13. doi: 10.1186/s40880-018-0289-9 (PMC5993144; doi:10.1186/s40880-018-0289-9)
Supplement: Supplementary file 6 — Additional file 6: Table S6. Genes associated with CKS2 expression level in pancreatic cancer tissues, based on data in The Cancer Genome Atlas. [file 40880_2018_289_MOESM6_ESM.docx]

Additional file 6: Table S6. Genes associated with *CKS2* expression level in pancreatic cancer tissues, based on data in The Cancer Genome Atlas

| Gene | Pearson Score |  | Gene | Pearson Score |  | Gene | Pearson Score |
| --- | --- | --- | --- | --- | --- | --- | --- |
| *CDKN3* | 0.82 |  | *CENPA* | 0.80 |  | *CDCA5* | 0.80 |
| *TPX2* | 0.80 |  | *FAM72B* | 0.80 |  | *PTTG1* | 0.79 |
| *ORC6* | 0.79 |  | *CDK1* | 0.77 |  | *UBE2C* | 0.77 |
| *RAD51* | 0.76 |  | *CCNB2* | 0.76 |  | *RPA3* | 0.75 |
| *TTK* | 0.75 |  | *ERCC6L* | 0.75 |  | *HJURP* | 0.75 |
| *KIF4A* | 0.75 |  | *CDC25C* | 0.74 |  | *E2F1* | 0.74 |
| *H2AFZ* | 0.74 |  | *AURKA* | 0.74 |  | *KIF2C* | 0.74 |
| *UBE2T* | 0.74 |  | *RACGAP1* | 0.74 |  | *BUB1* | 0.73 |
| *DLGAP5* | 0.73 |  | *KIF18B* | 0.73 |  | *CENPN* | 0.73 |
| *CDC45* | 0.72 |  | *RAD54L* | 0.72 |  | *AURKB* | 0.72 |
| *KIF23* | 0.72 |  | *TROAP* | 0.72 |  | *OIP5* | 0.72 |
| *CENPU* | 0.72 |  | *NUSAP1* | 0.72 |  | *PBK* | 0.72 |
| *BIRC5* | 0.71 |  | *CCNB1* | 0.71 |  | *RFC4* | 0.71 |
| *PRC1* | 0.71 |  | *SPAG5* | 0.71 |  | *AUNIP* | 0.71 |
| *CCT5* | 0.71 |  | *NCAPG* | 0.71 |  | *EXO1* | 0.70 |
| *KIAA0101* | 0.70 |  | *ZWINT* | 0.70 |  | *FAM64A* | 0.70 |
| *CEP55* | 0.70 |  | *ASF1B* | 0.70 |  | *SKA1* | 0.70 |
| *TICRR* | 0.70 |  | *MAD2L1* | 0.69 |  | *PLK1* | 0.69 |
| *FAM72D* | 0.69 |  | *PKMYT1* | 0.69 |  | *KIF20A* | 0.69 |
| *RMI2* | 0.69 |  | *FANCI* | 0.69 |  | *NCAPH* | 0.69 |
| *KPNA2* | 0.68 |  | *NEK2* | 0.68 |  | *TRIP13* | 0.68 |
| *BRIX1* | 0.68 |  | *DEPDC1* | 0.68 |  | *KNSTRN* | 0.68 |
| *POC1A* | 0.68 |  | *CCNA2* | 0.67 |  | *CDC6* | 0.67 |
| *RRM2* | 0.67 |  | *SHCBP1* | 0.67 |  | *PSRC1* | 0.67 |
| *NEIL3* | 0.67 |  | *KIF15* | 0.67 |  | *FEN1* | 0.66 |
| *MCM2* | 0.66 |  | *NUDT1* | 0.66 |  | *SMS* | 0.66 |
| *MELK* | 0.66 |  | *TRAIP* | 0.66 |  | *C17ORF53* | 0.66 |
| *EME1* | 0.66 |  | *DTL* | 0.66 |  | *TUBA1C* | 0.66 |
| *CKAP2L* | 0.66 |  | *PARPBP* | 0.66 |  | *POLA2* | 0.66 |
| *SKA3* | 0.66 |  | *CDC20* | 0.65 |  | *CDC25A* | 0.65 |
| *CKS1B* | 0.65 |  | *EZH2* | 0.65 |  | *ORC1* | 0.65 |
| *POLR2D* | 0.65 |  | *RANBP1* | 0.65 |  | *TK1* | 0.65 |
| *DSN1* | 0.65 |  | *MND1* | 0.65 |  | *SGOL1* | 0.65 |
| *BUB1B* | 0.64 |  | *KIF11* | 0.64 |  | *KIFC1* | 0.64 |
| *MYBL2* | 0.64 |  | *PCNA* | 0.64 |  | *SNRPB* | 0.64 |
| *TOP2A* | 0.64 |  | *GTSE1* | 0.64 |  | *RHEBL1* | 0.64 |
| *DNAJC9* | 0.64 |  | *CENPW* | 0.64 |  | *CHEK1* | 0.63 |
| *HMMR* | 0.63 |  | *HSPE1* | 0.63 |  | *TCF19* | 0.63 |
| *GINS1* | 0.63 |  | *NUF2* | 0.63 |  | *MCM10* | 0.63 |
| *DDIAS* | 0.63 |  | *LRR1* | 0.63 |  | *UBE2S* | 0.63 |
| *CCDC58* | 0.62 |  | *FANCB* | 0.62 |  | *MCM6* | 0.62 |
| *PSMA6* | 0.62 |  | *TCEB1* | 0.62 |  | *ARHGAP11A* | 0.62 |
| *KIF18A* | 0.62 |  | *CDCA2* | 0.62 |  | *CSTF2* | 0.61 |
| *ESPL1* | 0.61 |  | *STIP1* | 0.61 |  | *GINS4* | 0.61 |
| *GINS2* | 0.61 |  | *CENPF* | 0.60 |  | *RCC1* | 0.60 |
| *PSMA2* | 0.60 |  | *RFC2* | 0.60 |  | *XRCC2* | 0.60 |
| *MRPS30* | 0.60 |  | *C16ORF59* | 0.60 |  | *C5ORF34* | 0.60 |
| *SPC24* | 0.60 |  | *NCAPG2* | 0.60 |  | *PSMA4* | 0.59 |
| *SNRPA1* | 0.59 |  | *TXN* | 0.59 |  | *EIF2S2* | 0.59 |
| *CHAF1A* | 0.59 |  | *SMC4* | 0.59 |  | *POLQ* | 0.59 |
| *PLK4* | 0.59 |  | *GGCT* | 0.59 |  | *CYCS* | 0.59 |
| *ANLN* | 0.59 |  | *WDR62* | 0.59 |  | *CSE1L* | 0.58 |
| *DTYMK* | 0.58 |  | *H2AFX* | 0.58 |  | *KIF22* | 0.58 |
| *MKI67* | 0.58 |  | *PPIA* | 0.58 |  | *SNRPG* | 0.58 |
| *TYMS* | 0.58 |  | *ALG3* | 0.58 |  | *WDHD1* | 0.58 |
| *CHEK2* | 0.58 |  | *GSG2* | 0.58 |  | *SF3B6* | 0.58 |
| *ZWILCH* | 0.58 |  | *ENY2* | 0.58 |  | *MGME1* | 0.58 |
| *UHRF1* | 0.58 |  | *CENPH* | 0.58 |  | *CCT6A* | 0.57 |
| *FOXM1* | 0.57 |  | *POLE2* | 0.57 |  | *PSMA7* | 0.57 |
| *RFC5* | 0.57 |  | *RUVBL1* | 0.57 |  | *GLRX3* | 0.57 |
| *CBX3* | 0.57 |  | *EIF5AL1* | 0.57 |  | *CDT1* | 0.57 |
| *RACGAP1P* | 0.57 |  | *PSMG3* | 0.57 |  | *LSM5* | 0.57 |
| *MRPL13* | 0.57 |  | *MRPL17* | 0.57 |  | *ZNF367* | 0.57 |
| *EIF5A* | 0.56 |  | *PSMA5* | 0.56 |  | *TOMM5* | 0.56 |
| *RECQL4* | 0.56 |  | *RNASEH2A* | 0.56 |  | *NUP37* | 0.56 |
| *DSCC1* | 0.56 |  | *MTFR2* | 0.56 |  | *DIAPH3* | 0.56 |
| *DCUN1D5* | 0.56 |  | *CDCA4* | 0.56 |  | *MRPS22* | 0.56 |
| *ZNF695* | 0.56 |  | *MRPL47* | 0.56 |  | *ASPM* | 0.56 |
| *CCNF* | 0.55 |  | *RAN* | 0.55 |  | *SNRPB2* | 0.55 |
| *TPD52L2* | 0.55 |  | *AIMP2* | 0.55 |  | *FAM72A* | 0.55 |
| *EIF3B* | 0.55 |  | *DPM1* | 0.55 |  | *EIF4A3* | 0.55 |
| *PUM3* | 0.55 |  | *CENPO* | 0.55 |  | *UQCRHL* | 0.55 |
| *C20ORF24* | 0.55 |  | *CMC2* | 0.55 |  | *MRPL15* | 0.55 |
| *CENPK* | 0.55 |  | *FANCA* | 0.54 |  | *HELLS* | 0.54 |
| *FBXO22-AS1* | 0.54 |  | *RRM1* | 0.54 |  | *SNRPF* | 0.54 |
| *TUBA1B* | 0.54 |  | *PAIP1* | 0.54 |  | *DBF4* | 0.54 |
| *WDR76* | 0.54 |  | *PIF1* | 0.54 |  | *MRTO4* | 0.54 |
| *MED10* | 0.54 |  | *PHF5A* | 0.54 |  | *NANS* | 0.54 |
| *CDCA8* | 0.54 |  | *NOP10* | 0.54 |  | *C1ORF112* | 0.54 |
| *KIAA1524* | 0.54 |  | *CENPL* | 0.54 |  | *SNX8* | 0.54 |
| *PPIAL4C* | 0.54 |  | *CENPE* | 0.53 |  | *FANCD2* | 0.53 |
| *CENPI* | 0.53 |  | *SET* | 0.53 |  | *SIAH2* | 0.53 |
| *STIL* | 0.53 |  | *SNRPD3* | 0.53 |  | *SSBP1* | 0.53 |
| *UQCRH* | 0.53 |  | *TBRG4* | 0.53 |  | *KIF14* | 0.53 |
| *PSMD14* | 0.53 |  | *MIR4435-2HG* | 0.53 |  | *ZMYND19* | 0.53 |
| *PDZD11* | 0.53 |  | *POLE3* | 0.53 |  | *RRAS2* | 0.53 |
| *EXOSC2* | 0.53 |  | *SAPCD2* | 0.53 |  | *TMEM189* | 0.53 |
| *MTBP* | 0.53 |  | *BLM* | 0.52 |  | *HMGB2* | 0.52 |
| *MCM4* | 0.52 |  | *PSMA3* | 0.52 |  | *PSMD7* | 0.52 |
| *SEC13* | 0.52 |  | *TARS* | 0.52 |  | *BANF1* | 0.52 |
| *PPIH* | 0.52 |  | *AHSA1* | 0.52 |  | *DMC1* | 0.52 |
| *LINC00152* | 0.52 |  | *ARPC5L* | 0.52 |  | *BCL2L12* | 0.52 |
| *ATAD3A* | 0.52 |  | *RIBC2* | 0.52 |  | *PTTG3P* | 0.52 |
| *NXT1* | 0.52 |  | *CYC1* | 0.51 |  | *ECT2* | 0.51 |
| *HSPD1* | 0.51 |  | *PTMA* | 0.51 |  | *UCK2* | 0.51 |
| *HIST1H3J* | 0.51 |  | *HIST1H3B* | 0.51 |  | *RAE1* | 0.51 |
| *NCOA1* | -0.50 |  | *APOBEC3B* | 0.51 |  | *ARPC3* | 0.51 |
| *NUTF2* | 0.51 |  | *PAICS* | 0.51 |  | *RAI2* | -0.51 |
| *MESP2* | 0.51 |  | *MRPL37* | 0.51 |  | *COA4* | 0.51 |
| *OSER1* | 0.51 |  | *CEP68* | -0.50 |  | *RAD18* | 0.51 |
| *FTSJ1* | 0.51 |  | *ZDHHC15* | -0.50 |  | *BZW2* | 0.51 |
| *PSMC3IP* | 0.51 |  | *HNRNPC* | 0.50 |  | *PFDN4* | 0.50 |
| *PLP2* | 0.50 |  | *PRIM2* | 0.50 |  | *PSMB7* | 0.50 |
| *PSMD13* | 0.50 |  | *PSME2* | 0.50 |  | *VRK1* | 0.50 |
| *CENPP* | 0.50 |  | *PDCD5* | 0.50 |  | *EFTUD2* | 0.50 |
| *ANP32B* | 0.50 |  | *EBP* | 0.50 |  | *TMSB15A* | 0.50 |
| *DNAJB11* | 0.50 |  | *SGOL2* | 0.50 |  | *RALY* | 0.50 |
| *NIT2* | 0.50 |  | *EFCAB11* | 0.50 |  | *ESCO2* | 0.50 |
| *NTMT1* | 0.50 |  |  |  |  |  |  |
