# Supplementary figures and images for: Integrative analysis of gene expression profiles reveals specific signaling pathways associated with pancreatic duct adenocarcinoma
Source: Cancer Commun (Lond). 2018 Apr 27;38:13. doi: 10.1186/s40880-018-0289-9 (PMC5993144; doi:10.1186/s40880-018-0289-9)

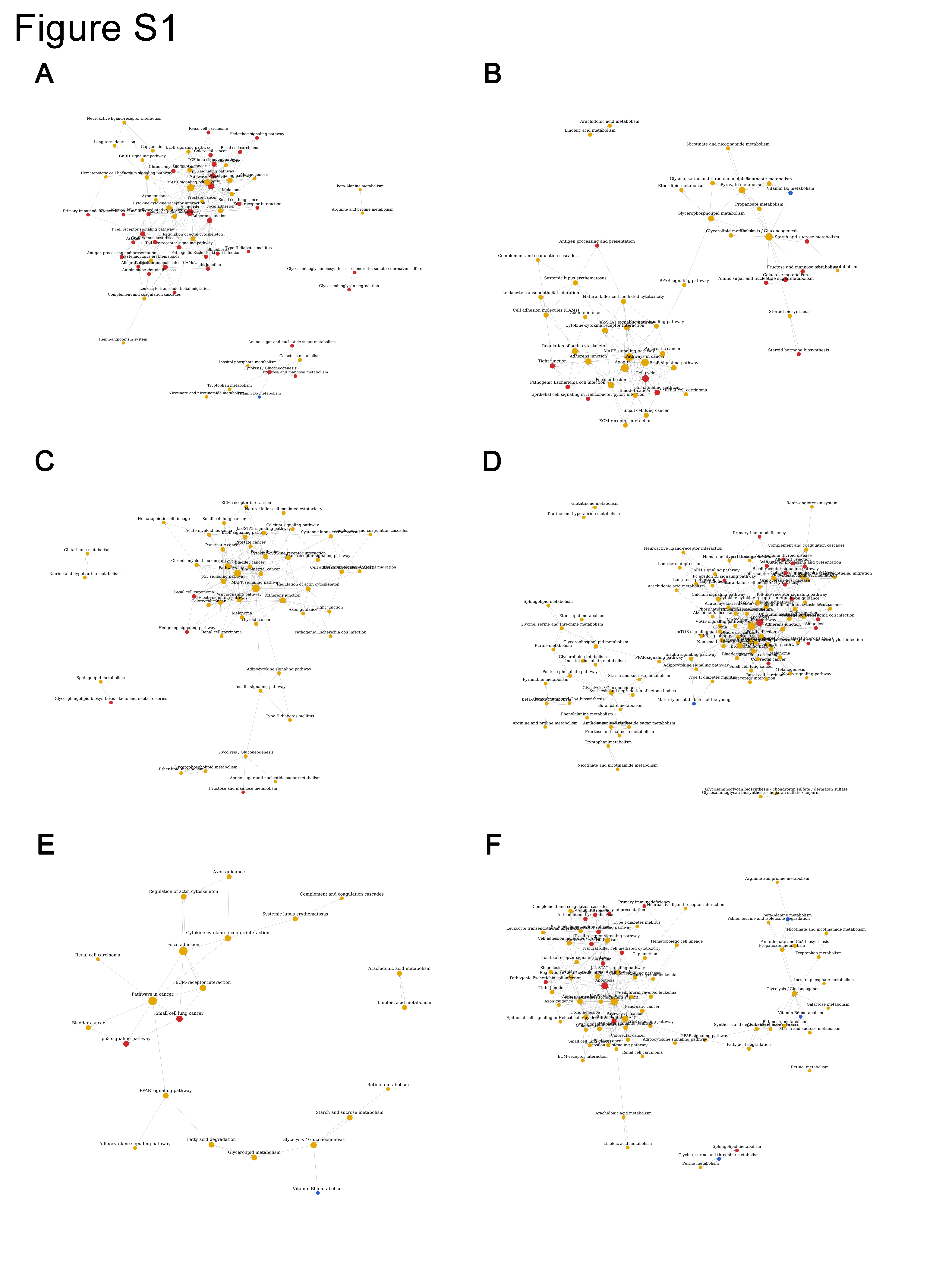

Supplement: Supplementary file 7 — Additional file 7: Figure S1. Pathway networks of all GEO datasets. Pathway networks for the datasets GSE15471 (A), GSE16515 (B), GSE32676 (C), GSE71989 (D), GSE28735 (E) and GSE42368 (F) were drawn using the GCBI online tool as described in Methods. Yellow dots indicate involvement of up- and down-regulated signaling pathway genes; red dots, up-regulated signaling pathway genes; and blue dots, down-regulated signaling pathway genes. The arrow points from the upstream toward the downstream signaling pathway. [file 40880_2018_289_MOESM7_ESM.tif]

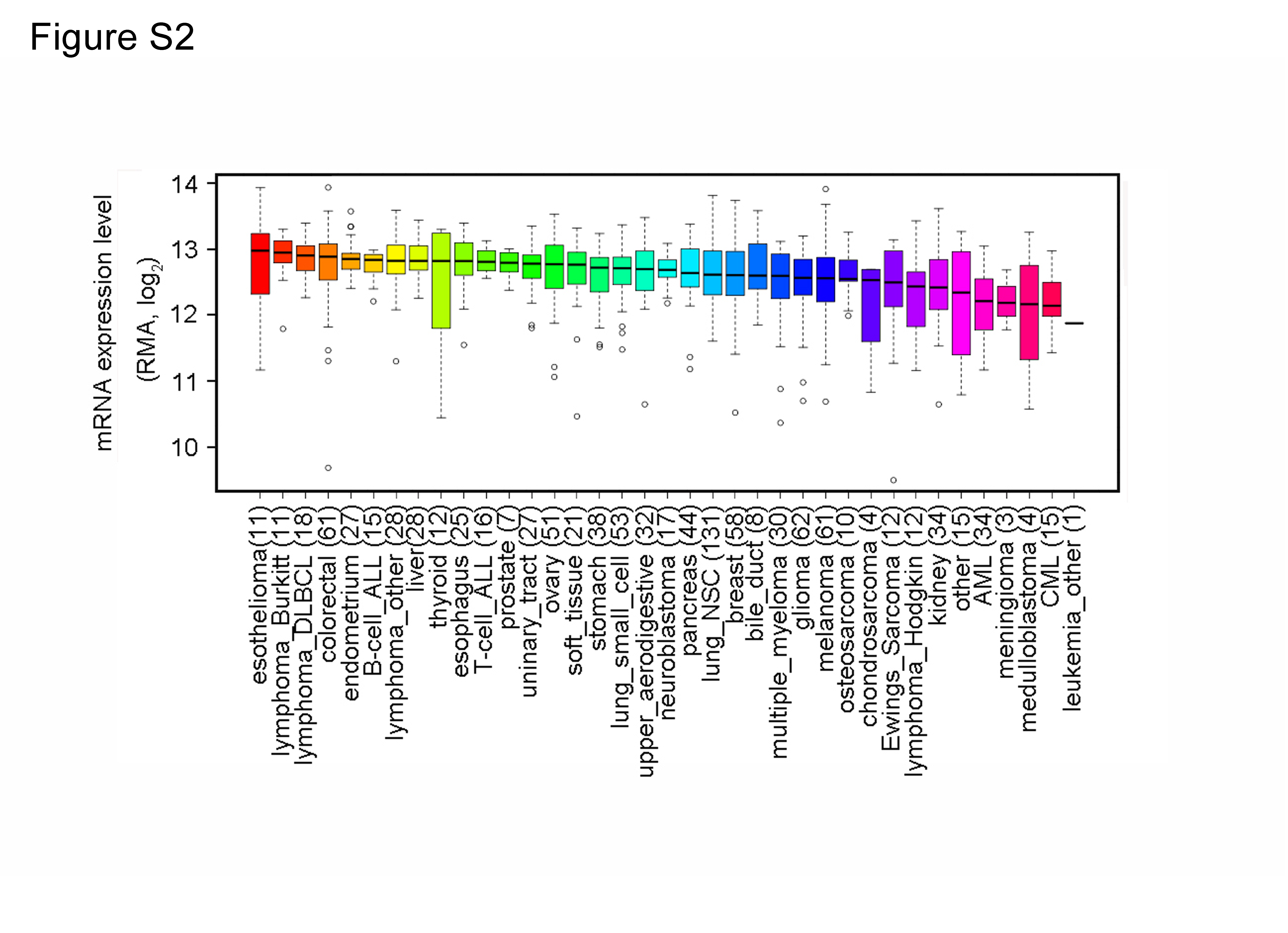

Supplement: Supplementary file 8 — Additional file 8: Figure S2. CKS2 expression in different types of cancer cell lines, based on data from the CCLE database (https://portals.broadinstitute.org/ccle/home). [file 40880_2018_289_MOESM8_ESM.tif]

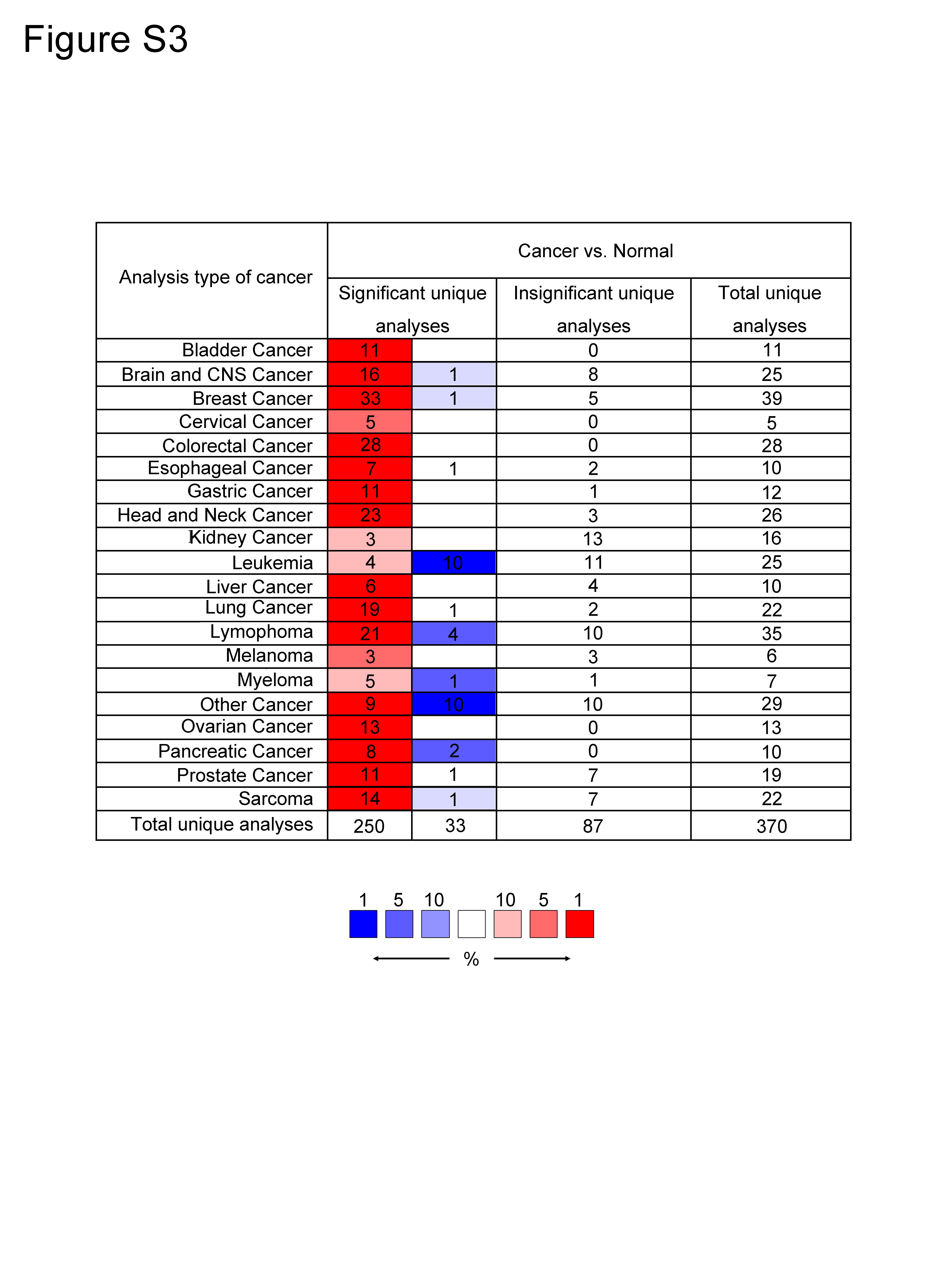

Supplement: Supplementary file 9 — Additional file 9: Figure S3. CKS2 expression across multiple types of cancer and corresponding normal tissues. The numbers of studies reporting up- or down-regulation is indicated, respectively, in red or blue boxes. Color intensity reflects the best gene rank percentile for analyses within the box. The following settings were used for the analysis: P=0.05, fold change=all, gene rank=all, and data type=mRNA (http://www.oncomine.org). [file 40880_2018_289_MOESM9_ESM.tif]
